# Supplementary material for: In-situ preservation of nitrogen-bearing organics in Noachian Martian carbonates
Source: Nat Commun. 2020 Apr 24;11:1988. doi: 10.1038/s41467-020-15931-4 (PMC7181736; doi:10.1038/s41467-020-15931-4)
Supplement: Supplementary file 1 — Supplementary Information [file 41467_2020_15931_MOESM1_ESM.pdf]

# Supplementary Information for

# In-situ preservation of nitrogen-bearing organics in Noachian Martian carbonates

**by Koike et al.**

## ■ Supplementary Notes

1. XANES spectra for the inorganic nitrogen reference materials

We obtained three N K-edge XANES spectra of the inorganic N-bearing references as Supplementary Fig. 1. Dinitrogen ($N_2$) has a strong sharp absorption peak at 400.8 eV due to its 1s → 1π* transition[1,2] with several smaller peaks around 406 – 409 eV. The vibrational manifold of the 1s→ 1π* transition was observed as the finely oscillating spectrum with our energy resolution power. Using this $N_2$ spectrum as a standard, we calibrated the photon energies of all the following XANES spectra.

Ammonium chloride ($NH_4Cl$) presents a sharp absorption peak at 400.8 eV with a broad feature around 405.5 eV. The 400.8 eV peak is identical to that of the $N_2$ π* resonance. This feature is attributed to the presence of interstitial $N_2$ molecules, which may be trapped and/or produced in ammonium salts.[2] The other feature at 405.5 eV is due to the 1s→ σ* transitions of $NH_4^+$ nitrogen.

Our XANES spectrum of $NaNO_3$ shows two sharp peaks at 401.4 eV and 405.2 eV, with a broad feature around 412.5 – 417.5 eV. The sharp peak at 405.2 eV is observed on nitrate salts in the previous studies,[1–3] while the lower energy peak at 401.4 eV is not observed in some conditions. Gillespie et al. (2008) attributed this 401.4eV absorption feature as the result of the X-ray-induced decomposition of nitrates. The 405.2 eV absorption is associated with 1s→ 1π* transitions of $NO_3$ nitrogen.[3]

2. XANES spectra for the organic nitrogen reference materials

XANES spectra of all the organic reference materials have a significant absorption feature at 398.9 eV (Supplementary Fig. 1). Almost all compounds, except for 2-aminoisobutyric acid and methionine, also present a significant feature at 399.9 eV. The spectrum of glycine shows an additional smaller absorption feature around 401.5 – 402.0 eV, which is much weaker in the spectrum of sarcosine. On the other hand, methylamine hydrochloride presents a strong sharp peak at 400.9 eV. Hydantoin also has a significant peak at 401.7 eV.

According to the literature, the feature at 398.9 eV is assigned to the π* resonance of the imino group[1] (Supplementary Table 2). Pyridinic N-heterocyclic groups also have a feature at a similar range of 398.7 – 400.7 eV. The diagnostic feature at 399.9 eV is due to the π* resonance of the nitrile group. Amide and amino groups have typical absorption features around 401.4 – 402.5 eV due to their 1s → 3p/s* transitions. All amino acid references measured in this study present weak but significant features around this energy range (Supplementary Fig. 1). The sharp peak at 400.9 eV of methylamine hydrochloride is due to either amine nitrogen or interstitial $N_2$ molecules. It is known that pyrrolic N-heterocyclic groups also have a significant and variable feature around 401 – 402.5 eV due to their π* resonance[1,3] (Supplementary Table 2). The strong 402.2 eV absorption of our N-heterocyclic compounds is due to their pyrrolic nitrogen.

3. XANES spectra for the scandium reference materials

We also measured the Sc $L_{II}$ and $L_{III}$-edge XANES spectra of $Sc_2O_3$, $ScCl_3{\cdot}6H_2O$, and $Sc_2(CO_3)_3{\cdot}H_2O$, in order to examine the possible interference to the nitrogen K-edge XANES.

All the Sc spectra demonstrate almost identical features; i.e., two pairs of sharp absorption peaks at 401.8 – 403.6 eV and 406.0 – 408.0 eV. Although the Sc $L_{III}$-edge XANES features appear in a similar energy range to the N K-edge features, the $L_{II}$-edge absorptions at 406.0 – 408.0 eV are distinct to the N XANES. Our results ensure that the Sc $L_{II}$-edge and $L_{III}$-edge XANES features are significantly different from the N *K*-edge XANES spectra, which means the interference effect of Sc on N XANES, if any, is negligible.

4. XANES spectra for the carbonates & silicate in ALH 84001 and the silver tape

We measured ALH carbonates (Crb-1 to 4) and silicate (Opx-1), as well as the silver tape during the two experimental periods in December 2018 and April 2019 (see Supplementary Table 1). Due to the December 2018 incident X-ray beam intensity being ~10 times higher than that in April 2019, the quality of the spectra of the former was better than the latter (i.e., higher signal-to-noise ratios for the December 2018 data; Supplementary Fig. 4). However, all the XANES spectra of the ALH carbonates show almost identical spectral shapes. They present two prominent absorption peaks at 398.9 eV and 399.9 eV with a broader absorption feature at around 408 eV. There are additional smaller peak(s) between 400.7 eV and 402 eV. Compared to the reference spectra described above, our ALH carbonates do not match the inorganic N-bearing compounds or the Sc-bearing compounds. Instead, they show similar features to those of the organic nitrogen reference materials. According to the literature,[1–3] the absorptions at 398.9 eV and 399.9 eV can be associated with imino group (398.7 – 398.9 eV), nitrile group (399.8 – 399.9 eV), and/or pyridinic N-heterocyclic groups (398.7 – 400.7 eV; summarized in Supplementary Table 2). The additional absorptions around 400.7 eV – 402 eV are in the same energy range as several N-

bearings groups, including pyrrolic N-heterocyclic, amide, and/or amino groups. Peaks corresponding to nitro group or urea group with absorption energies at 403 – 403.8 eV are not observed in our ALH carbonates. These results indicate that ALH carbonates contain significant amounts of the various N-bearing organic components, although the contributions from the inorganic components (i.e., $N_2$, $NH_4$, $NO_3$) or the Sc-bearing materials are negligible. In contrast, the XANES spectrum of ALH silicate (Opx-1) do not present clear peaks at the energy range of 398 eV – 399.5 eV. There is no visible peak observed at 400.7 eV – 402 eV, either. The XANES spectrum of the silver tape does not show any absorption peak in these energy ranges. Although these spectra indicate artificial local minimums around 403 eV – 404 eV, they do not affect the nitrogen signals.

## ■ Supplementary Discussion

1. Nitrogen speciation for ALH carbonates

Comparing our results of ALH carbonates and the organic reference compounds with the literature,[1] the plausible N-bearing organic groups can be estimated as: imino groups, nitrile groups, N-heterocyclic groups, amide, and/or amino groups, as well as macromolecular compounds containing these groups. It is uncertain whether all of these groups are in their original state or if some of them are decomposed secondarily due to X-ray beam damage. Previous studies mentioned that some organic matters are sensitive to X-ray irradiation, resulting in the formation of unsaturated N-bearing component (C=NH and C≡N) derivatives.[1,3] In our study, almost all the amino acid reference compounds present the significant absorptions corresponding to the imino and nitrile groups. It is likely that, under our XANES analytical conditions, most of the sensitive organic matter may be severely damaged by the incident beam. Although the imino and nitrile groups are observed in our ALH carbonates as well, some of them may be due to the X-ray beam damage of unknown precursors. Such interpretation is supported by the fact that the relative intensities between the two peaks at 398.9 eV (imino) and 399.9 eV (nitrile) of our ALH carbonates varied during the multiple repeated measurements (Supplementary Fig. 5).

2. Evaluation for the experimental contamination

As described in the Method section, we prepared our samples carefully to avoid the use of any organic resin or polishing pastes, as well as etching the surfaces of individual grains to depth of ~1 μm in depth using the FIB-SEM prior to the XANES analyses. We have explored the possibility of experimental contamination by measuring the silicate grain from

ALH 84001 (Opx-1) and a piece of blank silver tape. Because the igneous silicate parts of ALH 84001 should not contain Martian organic components, its XANES spectrum can be used to see the blank level of our measurements. On the other hand, the silver tape was measured to evaluate directly its likelihood of being a contaminant. If the XANES spectra of ALH Opx-1 and/or the silver tape had features similar to those of ALH carbonates, it could be inferred that our results were influenced severely by the experimental contamination during the sample preparations and/or the analytical processes. Compared to ALH carbonates, however, the absorption peaks at 398 eV – 399.5 eV are not clearly observed for ALH Opx without any visible peaks around 400.7 eV – 402 eV. The XANES spectrum of the silver tape does not have any absorption peak in these energy ranges. Although the silver tape spectrum presents an artificial local minimum around 403 eV – 404 eV, it does not affect the nitrogen signals. The absence of an absorption peak at around 398.9 eV – 401 eV indicates that the contribution from organic nitrogen is negligible for both the ALH Opx-1 and the silver tape. Consequently, the contamination of laboratory N-bearing organics during our experimental procedures is unlikely. It is inferred that the N-bearing species in ALH carbonates are either from Antarctic ice or from ancient Mars.

3. Evaluation for the Antarctic ice contamination

The origins of the organic components found in ALH 84001, i.e., either from Antarctica or from Mars, have been intensively discussed.[4–10] Based on the destructive chemical and isotopic analyses, the previous studies have suggested severe Antarctic contamination in ALH 84001.[6–8] Using the HPLC analysis of the hydrolyzed portions of the 1M-HCl extracts of the bulk rock fragments, Bada et al.[6] reported the presence of amino acids

in ALH carbonates with concentrations levels of up to several ppm levels. All of the detected amino acids were terrestrially common species (e.g., glycine, alanine, and serine) with the terrestrial-like L-rich enantiomeric ratios. Thus, they concluded that most were contaminants from Antarctic ice meltwater, although the absolute concentrations of the amino acids are significantly higher in ALH carbonates than in Antarctic ice (less than ppb). In principle, it is difficult to extract Martian indigenous organic components if they are mixed with Antarctic contaminants. In-situ or non-destructive analyses, however, may reduce the risk and degree of contamination and help to reveal Martian records. Based on the textural contexts of the hosting carbonates and magnetite in ALH84001, the previous in-situ observational studies[4,5,10] reported the probable presence of Martian reduced carbons, such as poly aromatic hydrocarbons (PAHs). The later TOF-SIMS imaging study[9] doubted their Martian origin. However, none of these previous *in-situ* studies focused on the N-bearing organic components in ALH 84001. The CN bond (e.g. C-N, C=N, or C≡N) was not observed by the previous C-STXM analyses,[5] probably due to their lower abundances compared to the C-C, C=C, and C-H bonds.

As described above, our ALH carbonates spectra indicate the presence of the organic N-bearing components and the apparent absence (or less significant amounts) of the inorganic N-bearing components. Oxidized nitrogen ($NO_x$) can be indicative of the contribution from Antarctic ice, which is expected to contain nitrate at ppm-level. $NO_x$-bearing aerosols could have been deposited and incorporated into the meteorite as well. Although we do not know the partition coefficient between $NO_x$ and carbonates, it is inferred that the incorporation of Antarctic materials into this sample may have been limited. It is indicated that the detected N-

bearing organic matters in ALH carbonates should be, at least partly, of Martian origin, although the possibility of Antarctic contamination cannot be completely excluded.

## Supplementary Figures

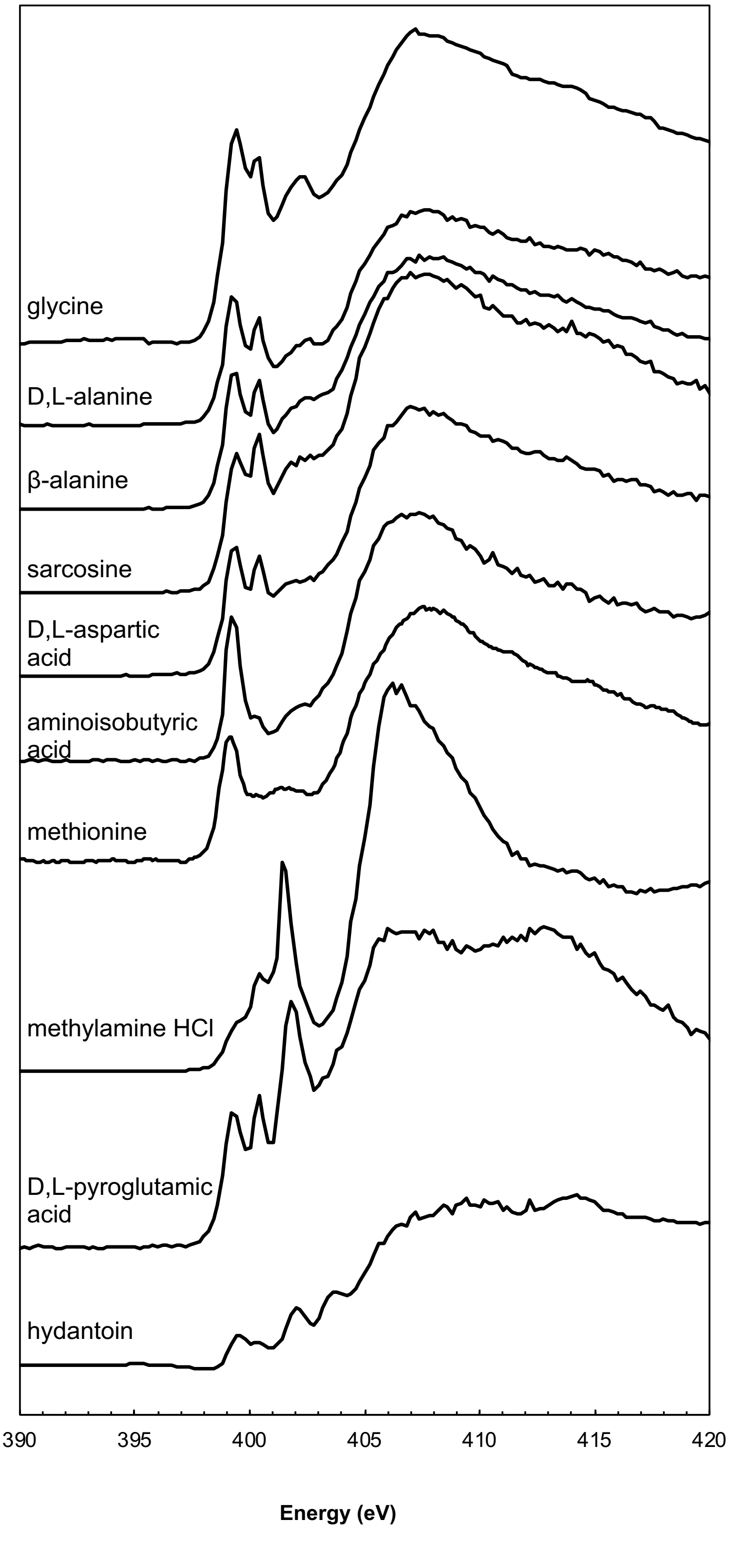

**Supplementary Fig. 1. Nitrogen K-edge XANES spectra of all organic N-bearing reference compounds.**

The analytical conditions of the individual grains are summarized in Supplementary Table 1. The original data for the all spectra is available in the separated Supplementary Table 3.

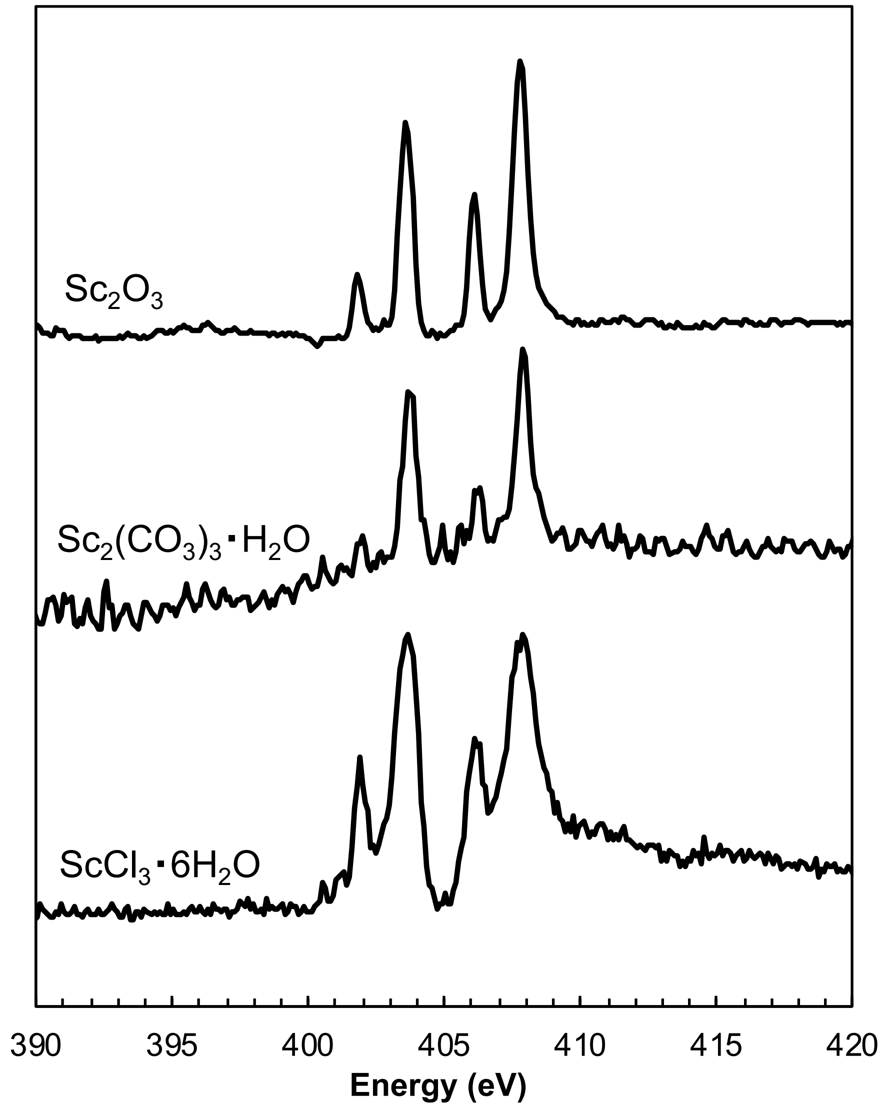


**Supplementary Fig. 2. Scandium $L_{II}$-edge and $L_{III}$-edge XANES spectra of the Sc-bearing reference compounds.**

The absorption energy range for Sc $L_{II}$-edge (406 – 408 eV) is clearly different from those of N K-edge absorptions, whereas Sc $L_{II}$-edge (402 – 404 eV) has an indistinguishable energy range. The original data for the all spectra is available in the separated Supplementary Table 3.

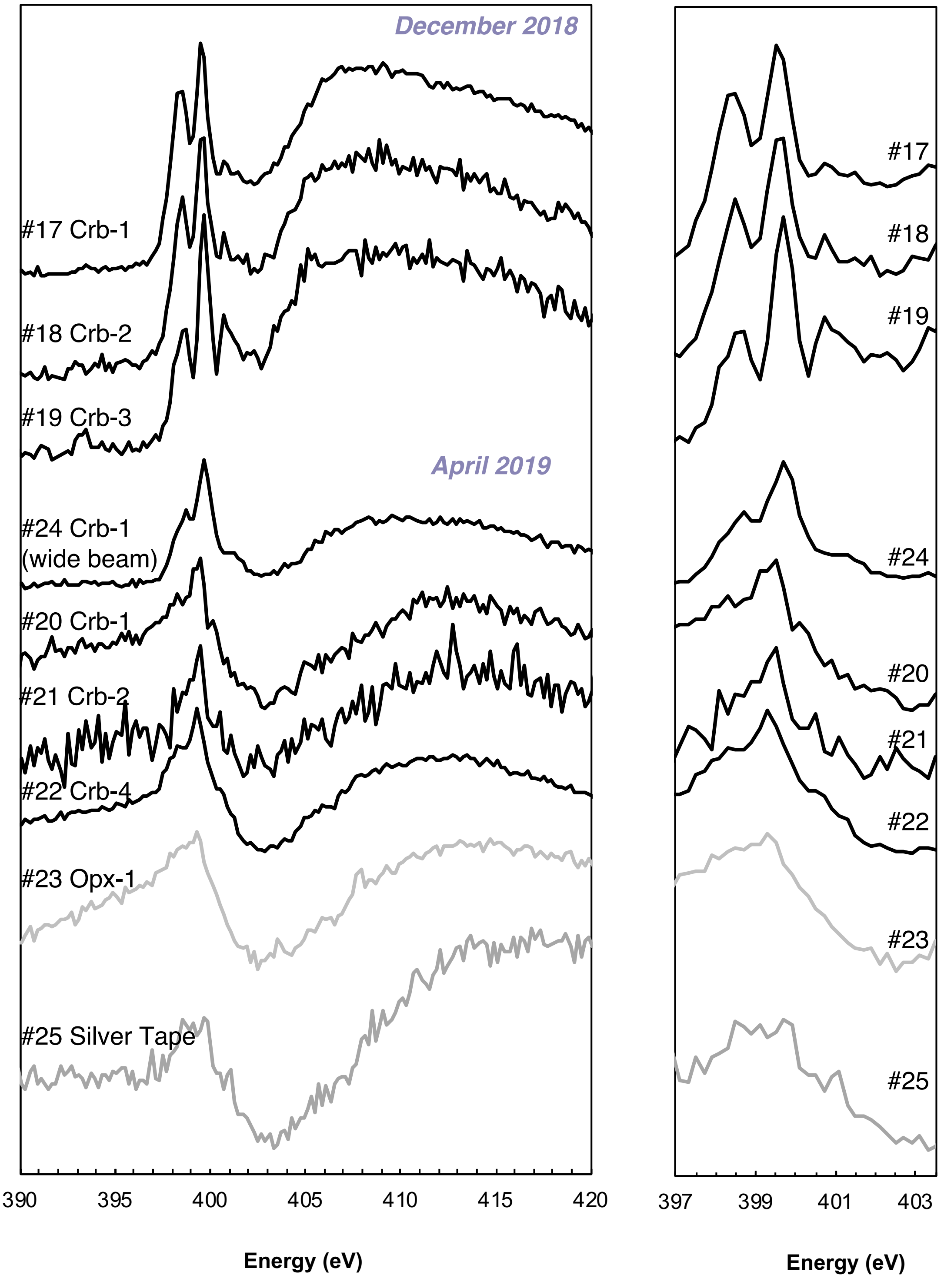
December 2018
April 2019
#17 Crb-1
#18 Crb-2
#19 Crb-3
#24 Crb-1
(wide beam)
#20 Crb-1
#21 Crb-2
#22 Crb-4
#23 Opx-1
#25 Silver Tape
390
395
400
405
410
415
420
Energy (eV)
#17
#18
#19
#24
#20
#21
#22
#23
#25
397
399
401
403
Energy (eV)

**Supplementary Fig. 3**. **XANES spectra of all ALH carbonates (Crb-1 to Crb-4), ALH silicate (Opx-1), and the silver tape.**

Labels are the same as in Supplementary Table 1. The upper 3 spectra (#17 - #19) were obtained during December 2018, the others were taken in April 2019. Left: The whole spectra. Right: The enlarged image of the energy range of 397 – 403.5 eV. The original data for the all spectra is available in the separated Supplementary Table 3.

a

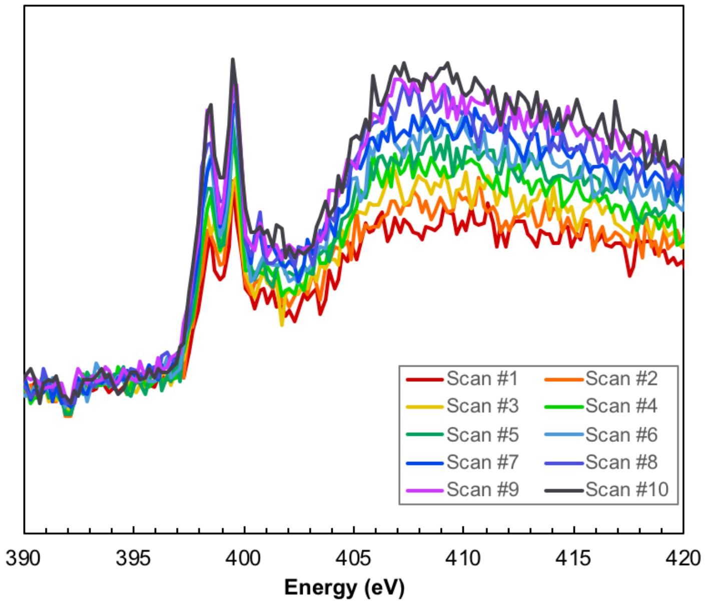


b

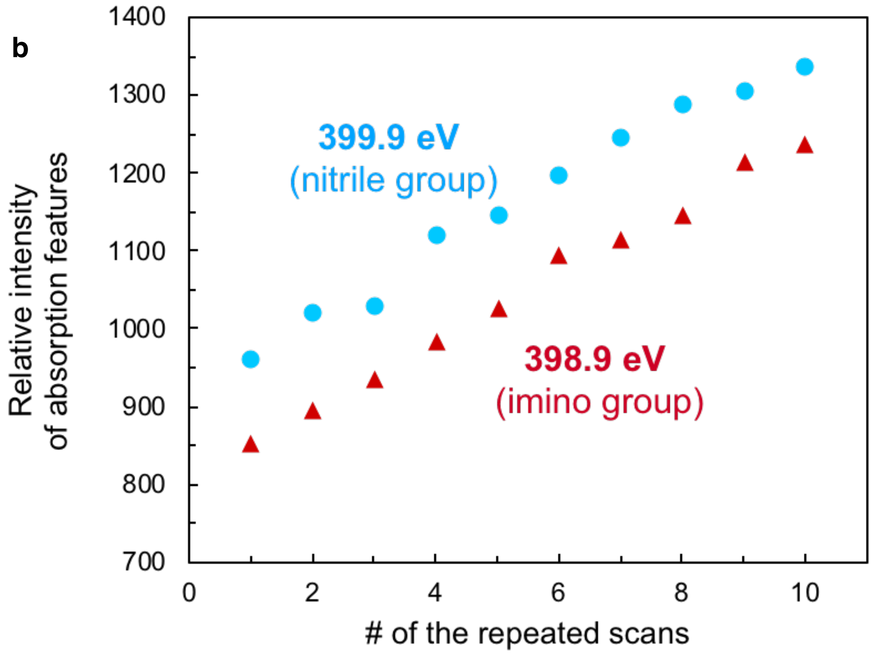


**Supplementary Fig. 4**. *(continued)*

c

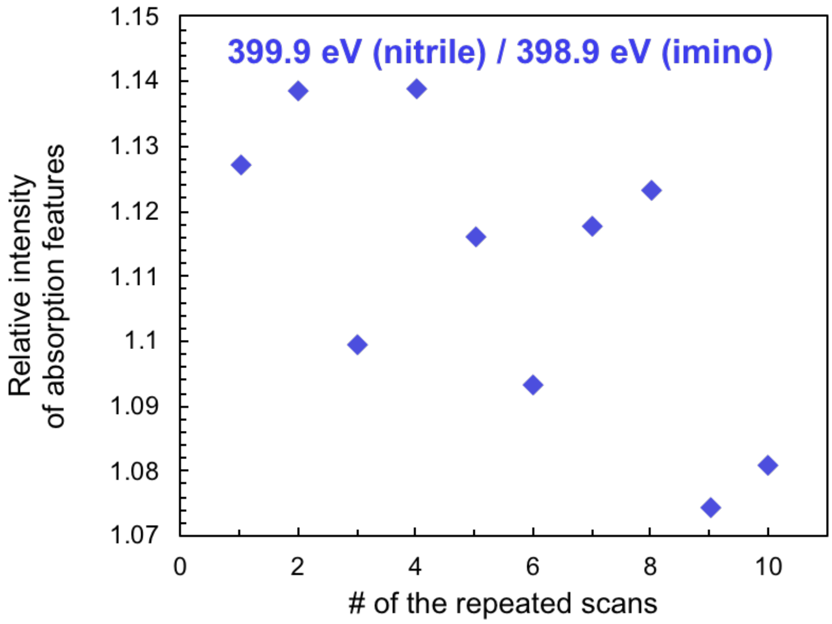


**Supplementary Fig. 4**. **Results of the repeated scans of ALH Crb-1, which are accumulated and plotted as a single spectrum in Fig. 2 of the main text.**

Absorption intensities of the two peaks at 398.9 eV (imino group) and 399.9 eV (nitrile group) vary during the multiple scans. **a,** Plots of the 10 spectra (before correction). **b & c,** Relationship between the number of repeated scans and individual peak intensities (**b**), and the relative intensities of 399.9 eV over 398.9 eV (**c**). The significant and systematic variations of the intensities indicate these imino- and nitrile-bearing compounds may have been produced during the XANES measurements, possibly due to X-ray damage of the precursor materials.

a

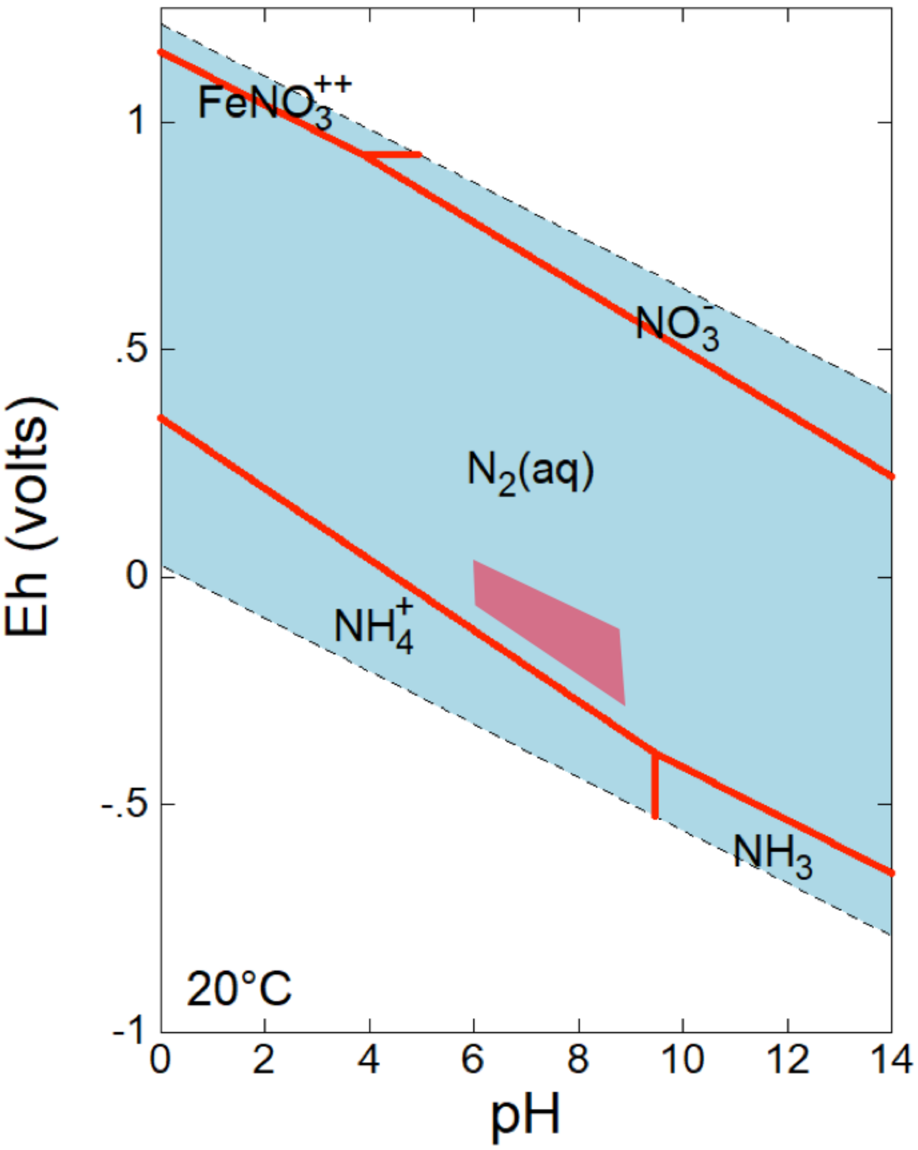


**Supplementary Fig. 5. Eh-pH conditions for nitrogen in the aqueous solution coexisting with the ALH carbonates at 4.0 Ga.**

**a,** $pCO_2$ = 0.1 bar, $NO_3^-$ = $10^{-5}$ M; **b**, $pCO_2$ = 0.1 bar, $NO_3^-$ = $10^{-10}$ M; **c,** $pCO_2$ = 0.1 bar, $NO_3^-$ = $10^{-20}$ M. Concentrations of the other elements are; Mg: $10^{-2}$, Fe: $10^{-6}$, Ca: $10^{-4}$, Mn: $10^{-2}$, Si: $10^{-3}$, Al: $10^{-11}$, K: $10^{-7}$, C: $10^{-2}$, S: $10^{-6}$ (mol/kg for all), modified after Kajitani et al.[11]

**b**

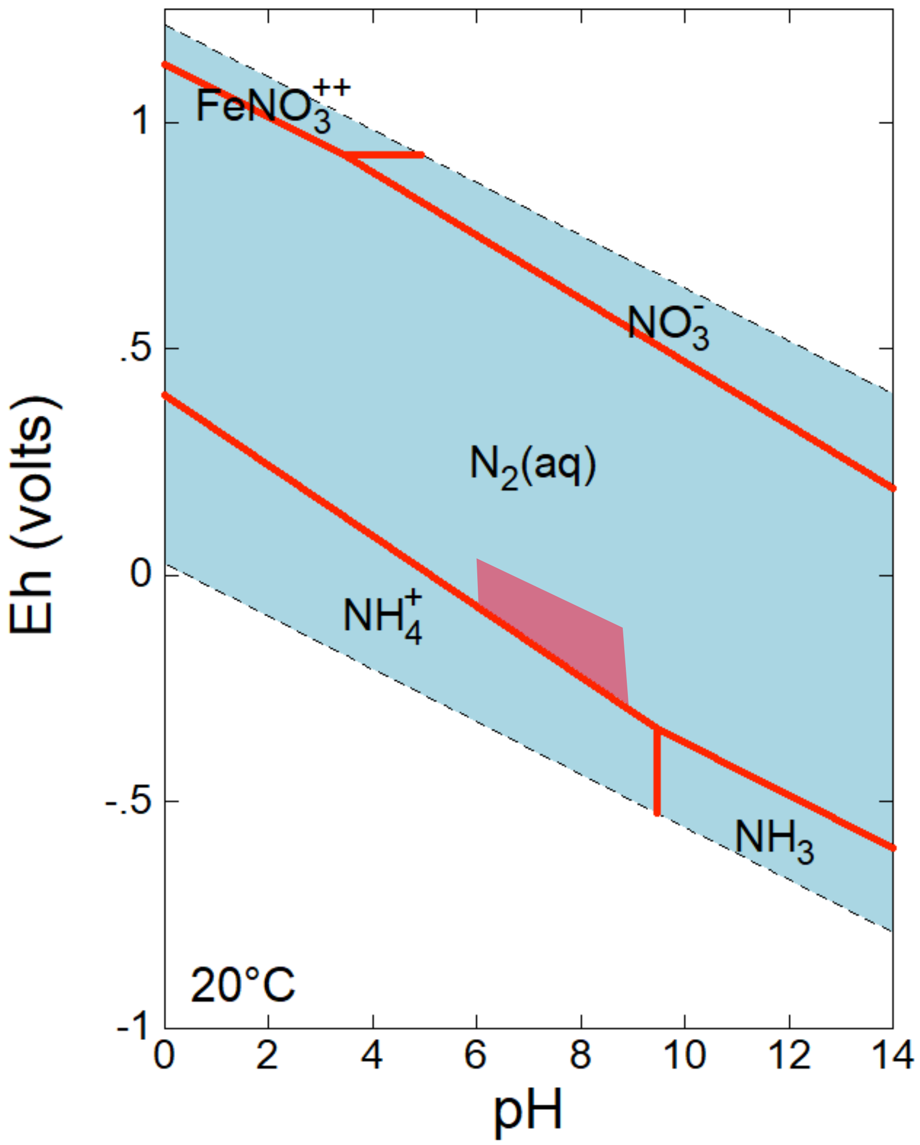


**Supplementary Fig. 5.** ***(continued)***

**c**

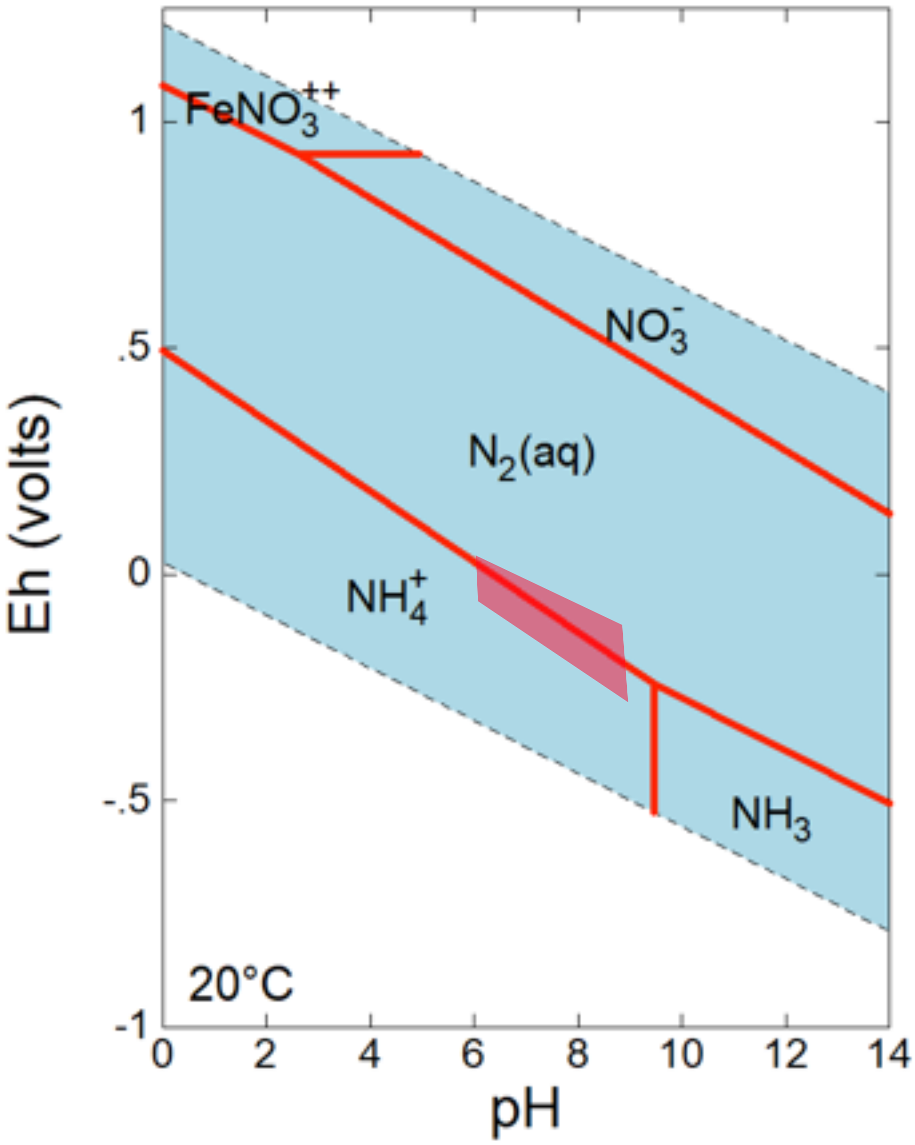


**Supplementary Fig. 5.** ***(continued)***

■ **Supplementary Table 1. The XANES analytical conditions of all reference materials and samples.**

| # | Sample name | Date | Type | beam size | Energy range (eV) | Energy step (eV) | Time /step (s) | # of repeated scans | Accum. time /step (s) | signal mode | beam size (μm) | Target |
|---|---|---|---|---|---|---|---|---|---|---|---|---|
| 1 | N2 gas | 12/2018 | Reference | wide | 400 - 417 | 0.02 | 1 | 1 | 1 | TEY | 10 (V) x 200 (H) | N2 |
| 2 | Ammonium chloride | 12/2018 | Reference | wide | 385 - 425 | 0.1 | 1 | 1 | 1 | TEY | 10 (V) x 200 (H) | NH4Cl |
| 3 | Sodium nitrate | 12/2018 | Reference | wide | 385 - 425 | 0.1 | 1 | 1 | 1 | TEY | 10 (V) x 200 (H) | NaNO3 |
| 4 | scandium(III) oxide | 12/2018 | Interreference | wide | 390 - 425 | 0.1 | 1 | 1 | 1 | TEY | 10 (V) x 200 (H) | Sc2O3 |
| 5 | scandium(III) carbonate hydrate | 12/2018 | Interreference | wide | 390 - 425 | 0.1 | 1 | 1 | 1 | TEY | 10 (V) x 200 (H) | Sc2(CO3)3·H2O |
| 6 | scandium(III) chloride hexahydrate | 12/2018 | Interreference | wide | 390 - 425 | 0.1 | 1 | 1 | 1 | TEY | 10 (V) x 200 (H) | ScCl3·6H2O |
| 7 | glycine | 4/2019 | Reference | wide | 385 - 425 | 0.2 | 15 | 1 | 15 | TEY | 10 (V) x 200 (H) | C2H4NO2 |
| 8 | DL-alanine | 4/2019 | Reference | wide | 385 - 425 | 0.2 | 15 | 1 | 15 | TEY | 10 (V) x 200 (H) | C3H7NO2 |
| 9 | β-alanine | 4/2019 | Reference | wide | 385 - 425 | 0.2 | 15 | 1 | 15 | TEY | 10 (V) x 200 (H) | C3H7NO2 |

| | | | | | | | | | | | | |
|---|---|---|---|---|---|---|---|---|---|---|---|---|
| 10 | N-methylamine (sarcosine) | 4/2019 | Reference | wide | 385 - 425 | 0.2 | 15 | 1 | 15 | TEY | 10 (V) x 200 (H) | C3H7NO2 |
| 11 | DL-aspartic acid | 4/2019 | Reference | wide | 385 - 425 | 0.2 | 15 | 1 | 15 | TEY | 10 (V) x 200 (H) | C4H7NO2 |
| 12 | 2-aminoisobutyric acid | 4/2019 | Reference | wide | 385 - 425 | 0.2 | 15 | 1 | 15 | TEY | 10 (V) x 200 (H) | C4H9NO2 |
| 13 | methionine | 12/2018 | Reference | wide | 385 - 425 | 0.1 | 1 | 1 | 1 | TEY | 10 (V) x 200 (H) | C5H11NO2S |
| 14 | methylamine hydrochloride | 4/2019 | Reference | wide | 385 - 425 | 0.2 | 15 | 1 | 15 | FY | 10 (V) x 200 (H) | CH5N·HCl |
| 15 | DL-pyroglutamic acid | 4/2019 | Reference | wide | 385 - 425 | 0.2 | 15 | 1 | 15 | TEY | 10 (V) x 200 (H) | C5H7NO2 |
| 16 | hydantoin | 4/2019 | Reference | wide | 385 - 425 | 0.2 | 15 | 1 | 15 | TEY | 10 (V) x 200 (H) | C3H4N2O2 |
| 17 | ALH Crb-1 | 12/2018 | Unknown | in-situ (l) | 385 - 425 | 0.2 | 15 | 10 | 150 | FY | 25 in diameter | carbonates |
| 18 | ALH Crb-2 | 12/2018 | Unknown | in-situ (l) | 385 - 425 | 0.2 | 15 | 4 | 60 | FY | 25 in diameter | carbonates |
| 19 | ALH Crb-3 | 12/2018 | Unknown | in-situ (l) | 385 - 425 | 0.2 | 15 | 2 | 30 | FY | 25 in diameter | carbonates |
| 20 | ALH Crb-1 | 4/2019 | Unknown | in-situ (s) | 385 - 425 | 0.2 | 3 s x 3 + 10 s x 4 | | 49 | FY | FY | carbonates |
| 21 | ALH Crb-2 | 4/2019 | Unknown | in-situ (s) | 385 - 425 | 0.2 | 10 | 3 | 30 | FY | 10 (V) x 30 (H) | carbonates |
| 22 | ALH Crb-4 | 4/2019 | Unknown | in-situ (s) | 385 - 425 | 0.2 | 10 | 10 | 100 | FY | 10 (V) x 30 (H) | carbonates |

| | | | | | | | | | | | | |
|---|---|---|---|---|---|---|---|---|---|---|---|---|
| 23 | ALH Opx-1 | 4/2019 | Contamination | in-situ (s) | 385 - 425 | 0.2 | 10 | 10 | 100 | FY | 10 (V) x 30 (H) | orthopyroxene |
| 24 | ALH Crb-1 | 4/2019 | Unknown | wide | 385 - 425 | 0.2 | 15 | 1 | 15? | FY | 10 (V) x 200 (H) | carbonates |
| 25 | Silver Tape | 4/2019 | Contamination | wide | 385 - 425 | 0.2 | 15 | 1 | 15 | FY | 10 (V) x 200 (H) | mixtures of organics and metal |

**Supplementary Table 2. Nitrogen K-edge XANES transition energies and the associated functional groups.** Data are from this study, Leinweber et al. [1], Gillespie et al. [2], and References therein.

| Energy range (eV) | transition | function group | |
|---|---|---|---|
| ***Inorganic materials*** | | | |
| 400.8 | 1s - π* | N2 (gas) | $N_2$ |
| 401.4 | 1s - π*? | nitrate & nitrite; possibly due to X-ray damage (?) | $M^+NO_3^-$ $M^+NO_2^-$ |
| 405.2 | 1s - π* | nitrate & nitrite | $M^+NO_3^-$ $M^+NO_2^-$ |
| 405 - 406 | 1s - σ* | ammonium | $NH_4^+X^-$ |
| 412.5 – 417.5 | 1s - σ* | nitrate | $M^+NO_3^-$ |
| ***Organic materials*** | | | |
| 398.7 - 398.9 | 1s - π* | imino | C=N |
| 398.7 - 400.7 | 1s - π* | pyridinic N-heterocyclic | 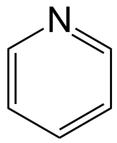 |
| 399.8 - 399.9 | 1s - π* | nitrile | C≡N |
| 401.0 - 402.5 | 1s - π* | pyrrolic N-heterocycles | 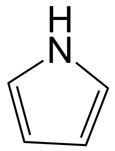 |

| | | | |
|---|---|---|---|
| 401.4 - 402.5 | 1s -3p/s* | amide & amino | $R{-}NH_X$ 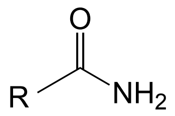 |
| 403.0 - 403.5 | 1s -3p/s* | urea | 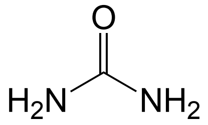 |
| 403.8 | 1s - π* | nitro | $R{-}NO_2$ |

**Supplementary References**


[1] Leinweber P. et al. Nitrogen K-edge XANES – an overview of reference compounds used to identify 'unknown' organic nitrogen in environmental samples. J. Synchrotron Rad. **14**, 500–511 (2007).

[2] Gillespie, A. W., Walley, F. L., Farrell, R. E., Regier, T. Z., and Blyth, R. I. R. Calibration method at the N K-edge using interstitial nitrogen gas in solid-state nitrogen-containing inorganic compounds. J. Synchrotron Rad. **15**, 532–534 (2008).

[3] Rodrigues F., Nascimento, G. M., and Santos, P. S. Studies of ionic liquid solutions by soft X-ray absorption spectroscopy. J. Electron Spectroscopy & Related Phenomena. **155**, 148–154 (2008).

[4] McKay, D. S. et al. Search for Past Life on Mars: Possible Relic Biogenic Activity in Martian Meteorite ALH84001. Science **273**, 924–930 (1996).

[5] Flynn, G. J., Keller, L. P., Miller, M. A., Jacobsen, C., and Wirick, S. Organic Compounds Associated with Carbonate Globules and Rims in the ALH 84001 Meteorite. 29th Lunar & Planet. Sci. Conf. (abstract) #1156 (1998).

[6] Bada J. L., Glavin, D. P., McDonald, G. D., and Becker, L. A Search for Endogenous Amino Acids in Martian Meteorite ALH84001. Science **279**, 362–365 (1998).

[7] Jull, A. J. T., Courtney, C., Jeffrey, D. A., and Beck, J. W. Isotopic evidence for a terrestrial source of organic compounds found in martian meteorites Allan Hills 84001 and Elephant Moraine 79001. Science **279**, 366–369 (1998).

[8] Becker, L., Popp, B., Rust, T., and Bada, J. L. The Origin of Organic Matter in the Martian Meteorite ALH84001. Adv. Space Res. **24**, 477–488 (1999).

[9] Stephan T., Jessberger, E. K., Heiss, C. H., and Rost, D. TOF-SIMS analysis of polycyclic aromatic hydrocarbons in Allan Hills 84001. Meteoritic. Planet. Sci. **38**, 109–116 (2003).

[10] Steele, A. et al. Comprehensive imaging and Raman spectroscopy of carbonate globules from Martian meteorite ALH 84001 and a terrestrial analogue from Svalbard. Meteoritic. Planet. Sci. **42**, 1549–1566 (2007).

[11] Kajitani, I. et al. Finding of oxidized sulfur species in carbonates from a martian meteorite Allan Hills 84001 using μ-XANES. The Ninth International Conference on Mars (abstract) #6446 (2019).
